# Annual Report on Midwifery

**Published:** 1846-06-01

**Authors:** 


### *Annual Report on Midwifery by* Dr. Warrington.

This is a highly interesting paper, presenting a digest of the developments in this department of the science which have characterised its history for the last few years. Some of these, as our readers need not be informed, are curious and importent. We shall make extracts from the Report, on some of the points which possess most interest.

*On the character of the tissue of the uterus.*—The following quotation is from a communication by *M. Jobert de Lamballe* in the *Journal de Chirurgie* (see also Am. Jour. Med. Sciences, Jan., 1845) on this topic.

"1. The proper tissue of the uterus in all animals, and at all periods, is not fibrous, but muscular.

"2. In pregnancy, the uterus is merely in a state of muscular hypertrophy.

"3. The uterus is formed by one muscle, and not by several.

"4. There exists an uterine mucous membrane, but it is without an epithelium.

"5. The direction of the uterine fibres shows how they act in freeing the organ of its contents. The longitudinal layers of fibres which originate at the fundus, and are inserted into the neck and vagina, tend to diminish the length of the uterus; whilst the semi-circular fibres, by their action diminish its cavity in every sense.

The longitudinal and annular fibres of the Fallopian tubes explain the mode of progression of the ovule in its passage from the ovary to the uterus; and those fibres which surround the uterine vessels appear to diminish by their contraction the rapidity of the circulation, and to prevent hemorrhage during parturition."

*On Reproduction.*—The new views on reproduction are especially interesting and not a little important. We extract a portion of the Report relating to this subject:

*On Reproduction.*—"Though numerous investigators, as Negrier, Paterson, Lee, Jones, and others, into the mode of reproduction in the zoological class of beings, have tended to modify the notions entertained by most physiologists for many years, yet it appears to have been reserved for Pouchet, Raciborski, and Bischoff, and perhaps for Duvernoy, to make a definite elucidation of this subject; and these latter gentlemen appear now to have brought the interesting question to an almost settled conclusion, both from analogy, and from a series of careful observations, not only on brutes, but in the human subject: that during the period of rutting in the former, and of menstruation in the latter, entirely independent of the application of the male sperm, an ovule escapes from the ovary, and is conducted through the Fallopian tube, uterus, and vagina; that preliminary to all this, a vesicle in the ovary enlarges, matures, and bursts, giving, first, exit to the ovule, and afterwards, receiving blood and lymph, passing through the various stages of absorption and cicatrization; thus exhibiting the yellow body in the ovary which had heretofore been regarded as a positive evidence of impregnation and conception. This view, of course, would, if generally acknowledged to be correct, demand a modification in our medico-legal opinious on this point. Still, that these opinions shall not be entirely reversed, it is acceded by some of these investigators, that the corpora lutea which occur after the escape of the ovules, and which have not been subsequently fecundated, are much smaller and less conspicuous than in cases in which the ovule has been retained in some part of the oviduct or uterus for subsequent developement, in consequence of impregnation.

" The conclusions of Pouchet, Professor of Zoology at the Museum of Natural History at Rouen, in his treatise, entitled "Positive theory of the Fecundation of the Mammiferæ, based upon observations made upon the entire animal series," published in Paris in 1842, are summed up in ten fundamental, and three accessory physological laws.

" 1. The human species forms no exception: the phenomena of its generation follow laws analogous to those which are observed among the different animals, and they are even perfectly identical with the acts which take place among those which occupy the head of the zoological series.

"2. Generation in all animals takes place by means of eggs, some inferior animals forming an exception.

" 3. In the whole animal series, the ovules pre-exist fecundation.

" 4. Physical obstacles prevent, in mammiferae, the possibility of the seminal fluid being placed in contact with the ovules while yet contained within the Graafian vesicles.

" 5. In the whole animal series, without the possibility of question, the ovary emits its ovules, independently of fecundation.

" 6. In all the animals, the ovules are emitted at periods fixed, and in relation with the increased periodical excitement of the genital organs.

" 7. In the mammiferæ, fecundation never occurs unless the emission of the ovules coincides with the presence of the seminal fluid.

"8. The emission of the catamenial discharge of women corresponds with the phenomena of excitement wnich manifest themselves at the period of the *amours* of the different beings in the zoological series, and especially of the females of the mammiferæ.

" 9. Fecundation offers a constant relation with the emission of the menses: thus, in the human species, it is easy to determine accurately, the inter-menstrual period at which conception is physically impossible, and that at which it may be probable.

"10. Asssuredly there exist no ovarian pregnancies, properly so called.

" 1st. Accessory law, fecundation in the mammiferæe is normally effected in the uterus.

" 2d. Abdominal and tubar pregnancies do not indicate that fecundation is normally effected in the ovary, and that it is this which causes the emission of the ovules.

" 3d. Normally, the Fallopian tubes only contract from within outwards, to transport the ovules.

*Discharge of the Liquor Amnii before the period of labor.*—Dr. W. quotes a case in which "the membranes ruptured and the liquor amnii escaped six weeks before the full period. The maternal abdomen become so flaccid that the parts of the fœtus could be distinguished through its parietes. The water was reproduced before delivery, which took place before the usual time." He also gives a case from his own experience, in which the membranes burst seven weeks before the full term of gestation. The discharge continued, a large number of napkins being daily saturated. Child delivered by forceps. "There was no appreciable quantity of liquor in the uterus during the time of labor."

*On the effects of prolonged Lactation and frequent child-bearing.*—We quote a portion of the Report relating to this subject. The circumstances mentioned are doubtless often causes of disease. We have frequent occasion to refer the origin of various neurotic disorders to these sources.

"The Edinburg Monthly Journal for April, 1845, quotes from Dr. Ashwell's work on diseases peculiar to females, some observations from that author, of special interest to the obstetric practitioner. Many of the consequences of undue lactation are to be met with in the range of business of the general practitioner, but the author of the book referred to, having had enlarged opportunities growing out of an extensive hospital and private practice amongst females, has been able to give a condensed view of the subject, well worthy the attentive perusal of all engaged in the exercise of the healing art. He says "functional amaurosis, accompanied by congestion of the conjunctiva is a very frequent result of excessive lactation, and seldom fails from its interference with the sight to arouse the patient's fears lest vision should be entirely and permanently lost."

Rapidly returning pregnancy, has been productive of equally severe consequences. I have, says Ashwell, known several instances, where during a pregnancy immediately succeeding the exhaustion from over-nursing, the eye has been almost constantly blood-shot, and the sight excessively imperfect. Specks and slight ulcerations of the cornea are occasionally connected with the exhaustion and irritability of nursing.—One case of each description alluded to has occurred under the observation of your reporter, during the past year. Dr. Ashwell considers that, in a great number of the cases, provided there be no serious organic lesion, the patient may be encouraged to expect to be restored, provided she abandon nursing, live easily, take nutritious diet, iron and quinine, pass much time in the open air, in the country, or by the sea side, though in some instances he acknowledges it may be necessary that she abstain from the use of her sight, and apply small blisters near the eyes. He also mentions some cases of jactitation, and a twitching, almost universal, throughout the extremities but especially about the face, dependent upon the same cause.

Epilepsy and insanity, the latter sometimes acute and well marked, though mostly of a mild kind, have both been observed by him as the consequence of undue lactation. His opinion of the pathology of these functional results of undue nursing, is that it consists in an attenuated and impaired condition of the blood, and a consequently depressed state of the nervous system, especially of the organic system of nerves."

